# A π-Configuration Plasmonic Dual Surface Plasmon Resonance Fiber Optic Sensor for Multi-Analyte Detection

**DOI:** 10.3390/s26123902

**Published:** 2026-06-19

**Authors:** John Ehiabhili, Radhakrishna Prabhu, Somasundar Kannan

**Affiliations:** School of Computing and Engineering Technology, Robert Gordon University, Aberdeen AB10 7GJ, UK; j.ehiabhili@rgu.ac.uk (J.E.); s.kannan1@rgu.ac.uk (S.K.)

**Keywords:** surface plasmon resonance, π-configuration, multi-analyte detection, metallic thin films, titanium dioxide (TiO_2_), refractive index sensing

## Abstract

Although optical fiber-based surface plasmon resonance (SPR) sensors have revolutionized real-time, label-free biosensing, conventional designs suffer from limited multi-analyte detection capabilities. This study utilizes the novel Pi (π)-configured dual SPR optical fiber sensor with two opposing side-polished surfaces, enabling plasmonic excitation for simultaneous multi-analyte detection. The proposed sensor leverages asymmetric metallic thin films such as Ag, Au, Cu, and hybrid configurations (metal + TiO_2_) to generate two distinct resonance peaks, significantly enhancing detection versatility. Numerical simulations using the finite element method in COMSOL Multiphysics v6.3 demonstrate that the π-configuration achieves dual resonance dips at 982 nm and 1276 nm for Ag and Ag–TiO_2_ films, 1040 nm and 1317 nm for Au and Au–TiO_2_ films, and 977 nm and 1249 nm for Cu and Cu–TiO_2_ films, respectively, for an analyte refractive index of 1.42. A peak spectral separation >125 nm was achieved for all the sensors for a refractive index range of 1.37–1.42, ensuring that the two dips are resolvable since the change in SPR wavelength is greater than or equal to the full width at half maximum, preserving dual-analyte capability and minimizing potential crosstalk. The results indicate that the π-configured dual SPR sensor utilizing silver and silver–TiO_2_ sensing layers had the highest wavelength sensitivity of 12,600 nmRIU^−1^ and 20,000 nmRIU^−1^, respectively, slightly outperforming its gold and copper counterpart. The optimized metallic and hybrid nanostructured films ensure dual distinct peaks with high sensitivity, while maximizing refractive index resolution. This work presents the design of a π-configured SPR-based optical fiber sensor utilizing dielectric and multi-metallic thin films, thereby offering a breakthrough in multiplexed biosensing for applications in medical diagnostics, environmental monitoring, and chemical detection.

## 1. Introduction

Surface plasmon resonance (SPR) sensors have emerged as a cornerstone of modern optical biosensing due to their label-free, real-time, specificity, and ultra-sensitive detection capabilities [1]. These sensors exploit the excitation of surface plasmon polaritons (SPPs) at the interface between a metal (typically gold or silver) and a dielectric medium, which is highly sensitive to changes in the refractive index (RI) of the surrounding medium [2]. The resonance condition is highly dependent on the optical properties of the metal film, the incident light wavelength, and the dielectric environment, making SPR an ideal platform for detecting biomolecular interactions, chemical analytes, and environmental pollutants [3]. Conventional Kretschmann prism-based SPR sensors have been widely used, but their bulky optics and high cost limit their applicability in point-of-care diagnostics and portable sensing systems [4]. In contrast, fiber optic SPR sensors offer a compact, flexible, and cost-effective alternative, with the added advantage of remote sensing capabilities [5,6]. Among these, D-shaped optical fiber SPR sensors have gained significant attention due to their enhanced evanescent field interaction resulting from side-polishing the fiber cladding, thereby improving sensitivity and detection limits [7]. Despite their advantages, conventional D-shaped SPR sensors suffer from two major limitations: the generation of only one resonance peak by most fiber SPR sensors, restricting their use to single-analyte detection, and limited sensitivity and crosstalk issues in multiplexed sensing: While some multi-analyte SPR sensors have been developed using spatial multiplexing (e.g., sensor arrays) [8] or wavelength/angular interrogation [9], these approaches require complex instrumentation, increasing cost and reducing robustness.

Recent attempts to enable multi-peak SPR sensing have included tilted fiber Bragg gratings (TFBGs) [10], but these suffer from fabrication complexity; multi-core fibers [11], which introduce crosstalk and signal interference; and bimetallic coatings, e.g., Au–Ag [12], but these still produce overlapping resonances unless carefully optimized. Thus, there remains a critical need for a simple, high-performance SPR sensor capable of generating two or more distinct resonance peaks for multi-analyte detection. To address these challenges, we propose a novel π-configured SPR-based optical fiber sensor [13], where two opposing side-polished surfaces are functionalized with asymmetric metallic and hybrid nanostructured thin films to generate two distinct resonance peaks. The key innovations of this work include a dual SPR sensor in π-configuration, optimized bimetallic and hybrid thin-film configurations, and COMSOL Multiphysics optimized design for minimal potential crosstalk and enhanced sensitivity.

Unlike traditional single D-shaped SPR sensors, our design features two polished surfaces on opposite sides of the fiber, enabling independent plasmonic excitation at two distinct locations. The π-configuration generates two independently resolvable resonance dips that can be individually monitored, and this architecture enables future dual-analyte detection when two different analyte environments are independently controlled. We first investigate identical metal films (Ag–Ag, Au–Au, Cu–Cu) to establish baseline performance. Next, we introduce hybrid configurations (e.g., Ag on one side and Ag–TiO_2_ on the other) to engineer two spectrally separated resonance dips. Titanium dioxide (TiO_2_) is selected due to its high refractive index (*n* ≈ 2.4–2.6), which enhances the RI contrast and plasmonic field confinement, leading to sharper resonance peaks [14]. A full-wave electromagnetic simulation is performed to analyze the electric field distribution, resonance wavelength shifts, and sensitivity for different thin-film configurations. The optimal metal thicknesses (Ag/Au/Cu: 45 nm, TiO_2_: 10 nm) [15,16] are determined to maximize sensitivity while minimizing damping losses. This study presents the first demonstration of a π-configuration SPR-based optical fiber sensor with asymmetric thin-film coatings (metal vs. metal–TiO_2_), which generates two distinct, spectrally separated resonance dips with significantly different sensitivities (up to 20,000 nm/RIU). This dual-channel architecture provides a foundation for future multi-analyte detection or self-referencing applications, where each sensing region can be independently functionalized and monitored.

The proposed sensor has broad applications in medical diagnostics for the simultaneous detection of multiple biomarkers, environmental monitoring for real-time sensing of heavy metals and organic pollutants in water, and chemical and gas sensing for the dual detection of volatile organic compounds (VOCs). By enabling multiplexed sensing in a single fiber, this work eliminates the need for complex sensor arrays while maintaining high sensitivity and low crosstalk. This work bridges a critical gap in SPR sensing by enabling simultaneous detection of two analytes without complex instrumentation, paving the way for next-generation biosensors. Future work will explore experimental validation and on-chip microfluidic integration.

## 2. Sensor Structural Design and Theoretical Simulations

### 2.1. Sensor Geometry and Material Configuration

**Figure 1:**
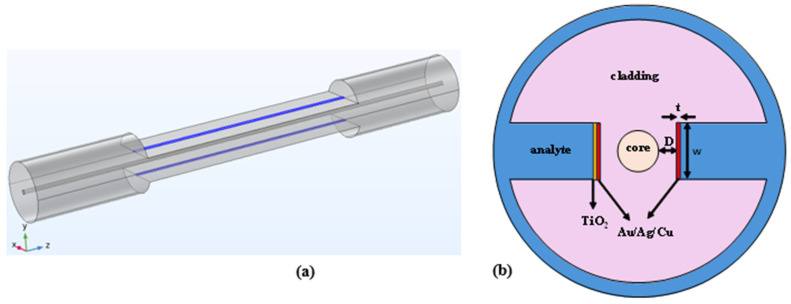
(**a**) 3D schematic of π-configuration SPR sensor; (**b**) sensor design in 2D.

The proposed π-configured optical fiber SPR sensor consists of a standard single-mode fiber (SMF-28, Corning, LA, USA) with two opposing side-polished regions to create dual sensing regions. This geometrical design was carried out using COMSOL Multiphysics software(v6.3). It consists of a 3.1% GeO2 doped silica core with diameter 9 µm, and a pure silica cladding with diameter 125 µm. Figure 1a shows the 3D schematic diagram of the π-configuration SPR-based fiber optic sensor with dual sensing regions coated with metallic thin films. The proposed sensor can be fabricated through the side-polishing technique (mechanical polishing) [17] or laser micro-machining [18]. This technique partially removes the cladding on opposite sides to expose the evanescent field while preserving the fiber’s mechanical integrity, creating a flat polished surface on opposite sides of the fiber that allows uniform deposition of plasmonic films and dielectric layers. The amount of residual cladding, D, for each of the sensing regions is 0.7 µm from the core of the fiber. This has been carefully optimized to maximize evanescent field interaction while minimizing leakage losses. Magnetron sputtering or thermal evaporation techniques are utilized for the plasmonic thin-film deposition on the dual planar sensing regions [19,20]. The width w of the plasmonic sensing regions were optimized at 10 µm, while the thicknesses $t_{m}$ and $t_{t}$ of the metallic thin-film and titanium dioxide dielectric, respectively, were optimized at 45 nm and 10 nm, respectively [15]. The 2D π-configuration dual SPR-based fiber optic sensor cross-sectional schematic shows the fiber after two opposing side-polishing steps to create flat surfaces, leaving a rectangular-like residual cladding profile, with the design parameters illustrated in Figure 1b.

### 2.2. Simulation Setup

The numerical simulations were conducted using COMSOL Multiphysics 6.3, employing the wave optics module to solve Maxwell’s equations in the frequency domain using the electromagnetic wave frequency-domain (ewfd) module because our sensor operates under continuous-wave excitation. We are interested in steady-state field distribution and wavelength-dependent transmission, making the frequency-domain formulation computationally efficient and physically appropriate. The computational domain consists of the fiber core and cladding refractive index from the Sellmeier equation, metallic thin films (Ag, Au, Cu) of thickness 45 nm, residual cladding thickness of 0.7 µm, and dielectric (TiO_2_) thickness of 10 nm modeled using experimentally validated Drude–Lorentz dispersion relations, operating wavelength of 600–1200 nm, and analyte region with refractive indices ranging from 1.37 to 1.42.

A perfectly matched layer (PML) was incorporated at the boundaries of the simulated region to eliminate unwanted reflections of any incident waves. The meshing strategy employed a physics-controlled extra-fine triangular mesh with a maximum element size of λ/10 in the core and λ/5 in the metal layers, refined at metal–analyte interfaces to accurately capture plasmonic wave propagation. For the experimental setup of a π-configured dual SPR fiber optic sensor for in vitro applications, the proposed sensor could be used for the simultaneous detection of two cardiac biomarkers, such as troponin I and B-type natriuretic peptide (BNP), from a single serum sample. One sensing region may be functionalized with antibodies against troponin I and the other against BNP, enabling rapid point-of-care diagnosis of acute myocardial infarction and heart failure. Alternatively, the sensor can be reconfigured for an analyte flow mechanism. The π-configured sensor is embedded in a microfluidic flow cell with narrow channels that allows controlled analyte flow directly over the exposed sensing regions. The flow cell could be made of glass or polydimethylsiloxane (PDMS) to ensure optical transparency for light coupling into the fiber [21]. A syringe pump could be used to inject the analyte at controlled speeds. A broadband light source such as a tungsten-halogen lamp could then be coupled into the fiber sensor, and a spectrometer which captures the transmission spectrum could analyze the shift in SPR dip based on the analyte refractive index. For this research, the desired analytes are viruses; however, other analytes like bacteria, glucose, proteins, and heavy metals in solutions may be used. Analytes interact with the sensor by varying its refractive index at the sensor surface, which simulates the binding of biomolecules. As a result, molecular interactions can be simulated and the sensitivity of the sensor assessed. To evaluate the performance of the sensor, parameter studies are conducted under various operating conditions. The effects of varying wavelengths, refractive indices, and geometric parameters on the SPR response are investigated by sweeping through the range of wavelengths, refractive indices, and geometric parameters. By using simulations, the optimal sensor design and operational parameters can be identified.

In SPR-based fiber optic sensors, resonance occurs when the wavevector of the guided optical mode in the fiber matches the wavevector of SPs at the metal–dielectric interface, ensuring efficient energy transfer from the incident light to the SPWs, leading to a characteristic dip in transmission. At this interface, SPs propagate with a wavevector $k_{SPP}$, given by [22]

(1) $$k_{SPP}=\frac{2\pi }{\lambda }\sqrt{\frac{\varepsilon_{m}\varepsilon_{d}}{\varepsilon_{m}+\varepsilon_{d}}}$$

where λ is the wavelength of incident light and $\varepsilon_{m}$ and $\varepsilon_{d}$ are permittivity of the metal thin film and the dielectric analyte, respectively. The wavevector $k_{mode}$ of the fundamental guided mode of an optical fiber is given as [23]

(2) $$k_{mode}=\frac{2\pi }{\lambda }n_{eff}$$

where $n_{eff}$ is the effective index of the guided mode in the fiber. It depends on the fiber core, cladding, and metal thin film layer, and is obtained by solving the wave equation for optical fiber modes. The phase matching condition requires that $k_{mode}{\;=\;k}_{SPP}$ for resonance to occur [24]. Thus,

(3) $$n_{eff}=\sqrt{\frac{\varepsilon_{m}\varepsilon_{d}}{\varepsilon_{m}+\varepsilon_{d}}}$$

Equation (3) determines the refractive index at which SPR occurs based on the fiber mode properties, metal thin film type, and analyte refractive index.

The refractive indices of the Ge doped core and the silica cladding are determined by utilizing the Sellmeier equation [25]:

(4) $$n^{2}\left(\lambda \right)=1+\frac{A_{1}\lambda^{2}}{\lambda^{2}-B_{1}}+\frac{A_{2}\lambda^{2}}{\lambda^{2}-B_{2}}+\frac{A_{3}\lambda^{2}}{\lambda^{2}-B_{3}}$$

where λ is the wavelength of incident light in μm, n is the fiber core or cladding wavelength-dependent refractive index, and $A_{1}$, $A_{2}$, $A_{3}$, $B_{1}$, $B_{2}$, and $B_{3}$ are Sellmeier’s constants obtained from [25].

Using the Drude–Lorentz model, the metal thin film dielectric function is determined by the relation [26]

(5) $$\varepsilon_{m}\left(\omega \right)=\varepsilon_{\alpha }-\frac{\omega_{P}^{2}}{\omega^{2}+i\gamma \omega }+∆\varepsilon_{P}\frac{\Omega_{P}^{2}}{\Omega_{P}^{2}-\omega^{2}-i\Gamma_{P}\omega }$$

where $\omega_{P}$ is the plasma frequency, $\varepsilon_{\alpha }$ is the metal interband offset, γ is the damping coefficient, $∆\varepsilon_{P}$ is the weighting coefficient, ${Ω}_{P}$ is the oscillator strength, and $\Gamma_{P}$ is the spectral width. The values of the Drude–Lorentz parameters for Ag, Au, and Cu are obtained from [27].

We consider a dielectric layer of ${TiO}_{2}$ of thickness $t_{d}$ and permittivity $\varepsilon_{d}$ between the metal and analyte, where it is assumed that the metal is thick enough to be semi-infinite in the x direction, and field decays away from the interfaces. Approximating for a thin, high index dielectric, ${TiO}_{2}$, we obtain the modified phase-matching condition governed by the implicit equation derived from the transverse resonance condition for the asymmetric three-layer structure given as [24]

(6) $$\kappa_{d}tanh(\kappa_{d}t_{d})=\frac{\varepsilon_{d}\left(\frac{\kappa_{m}}{\varepsilon_{m}}\right)+\varepsilon_{a}\left(\frac{\kappa_{a}}{\varepsilon_{a}}\right)}{1+\frac{\varepsilon_{a}\kappa_{m}}{\varepsilon_{m}\kappa_{a}}}$$

where $\kappa_{d}$ is the inverse of the transverse decay length in ${TiO}_{2}$, tanh is a hyperbolic tangent that describes the confinement of the field in thin dielectric layers, $\kappa_{m}$ and $\kappa_{a}$ are transverse wavevectors in the metal and analyte, respectively, and $\varepsilon_{a}$ is the permittivity of the analyte. The term on the RHS embodies the modification of the effective surface plasmon boundary conditions by the presence of the ${TiO}_{2}$ layer, shifting the resonance relative to the metal–analyte interface.

The refractive index (*n*) of ${TiO}_{2}$ can be calculated using the expression [28]

(7) $$n^{2}=5.913+\frac{0.2441}{\lambda^{2}-0.0803}$$

The transmission coefficient (T) provides crucial information about the interaction between the light and sensing medium over a range of wavelengths [29]. It is expressed as

(8) $$T=exp\left(\frac{4\pi }{\lambda }\cdot {\left(n_{eff}\right)}_{imag}\cdot L\right)$$

where ${\left(n_{eff}\right)}_{imag}$ is the imaginary part of the effective refractive index and L is the sensing region length. In SPR sensors, sensitivity S is the shift in the resonance wavelength ($∆\lambda_{res}$) per unit change in the analyte refractive index ($∆n_{a}$). It is given as [30]

(9) $$S=\frac{∆\lambda_{res}}{∆n_{a}}$$

The figure of merit (FOM) is a crucial parameter that quantifies the sensor’s ability to distinguish between different analyte RIs based on the sharpness and depth of the transmission spectrum dip. Typically, it is expressed as [31]

(10) $$FOM=\frac{S}{FWHM}$$

FWHM is the full width at half maximum of the SPR resonance dip, and it is a measure of the sharpness of the resonance [32].

## 3. Results and Discussions

### 3.1. Comparing Sensitivities of π-Configuration and D-Shaped SPR Sensors

The electric field of SPR-based fiber optic sensing is advanced by leveraging identical metal thin films in a dual sensing region π-configuration, which offers a unique advantage of isolating the geometric contribution to crosstalk (opposite-side polishing) from material effects. This provides a baseline for the different hybrid configurations as sensing regions. The sensitivity for the π-configuration sensor using the same metal (Ag–Ag, Au–Au, and Cu–Cu) on both sensing surfaces is compared to that of a D-shaped optical fiber SPR-based sensor. Table 1 shows the comparison of sensitivities of the proposed π-configured dual SPR sensor using same combination of Ag/Au/Cu metallic thin films and the D-shaped SPR sensor using Ag/Au/Cu thin films from the recent literature [15].

At a high RI (n_a_ = 1.42), sensitivity is primarily governed by metal–dielectric contrast, not fiber geometry. Since both sensors use identical metallic thin films (45 nm), their plasmonic responses are inherently similar. At n_a_ = 1.42, the evanescent field penetration depth (~200 nm for Ag) approaches its maximum interaction limit. The pi-configuration’s second sensing zone adds minimal extra field overlapping with the analyte.

The key advantage of the π-configuration is the dual-peak multiplexing capability. Unlike a single D-shaped sensor with one resonance peak, the π-configuration generates two distinct peaks if asymmetric coatings are used such as metal vs. metal + dielectric. This enables the simultaneous detection of two analytes without crosstalk. Another advantage of the π-configuration is that opposite-side polishing minimizes evanescent field overlapping between zones. This reduces interference when detecting dissimilar analytes. Also, dual sensing points allow for self-referencing, where one region monitors the reference RI, and the other detects the analyte. This helps to improve reliability in noisy environments such as those with temperature fluctuations.

### 3.2. Investigating π-Configuration Sensor Using Bimetallic Asymmetry Material

The π-configuration dual spatially separated sensing interface provides a unique architectural opportunity for multi-analyte detection using bimetallic asymmetry material. This method uses noble metals’ material-specific dispersion to generate unique plasmonic resonances from the two sensing regions of the π-configuration [33]. The first sensing region was functionalized with a 45 nm silver (Ag) film, whereas the second sensing region was functionalized with a 45 nm gold (Au) film. This is a bimetallic π-sensor with the plasmonic metal’s complicated dielectric function, $\varepsilon_{m}\left(\omega \right)$, as the only variable between the regions.

**Figure 2:**
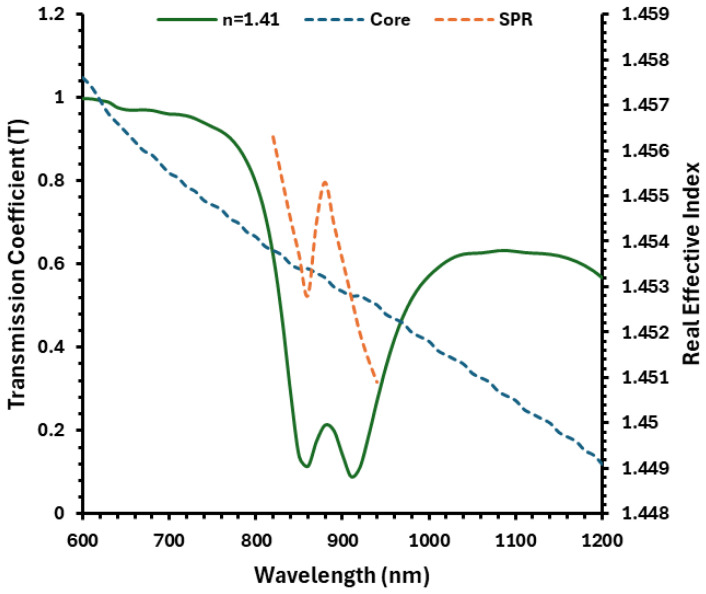
Phase matching between core and SPP modes of a bimetallic asymmetry π-configuration sensor.

The operating principle is based on the unique dispersion relations between Ag and Au SPP modes. Figure 2 shows the transmission spectrum of the phase match between the core and SPP modes of the bimetallic asymmetric π-configuration sensor at an analyte RI of 1.41. This gives the real part of the effective index $\left(Re\left(n_{eff}\right)\right)$ as a function of wavelength for the fiber core mode (HE_11_) and the consequent hybridized SPP modes for each metal at an RI of 1.41. For this RI, the Ag-region has a resonant wavelength of 856 nm, while the Au-region has a larger resonant wavelength of 912 nm. The intrinsic spectral separation (∆λ = 56 nm) creates a natural demultiplexing mechanism, allowing the two resonance dips to be resolved within a single transmission spectrum without complex optical filtering.

**Figure 3:**
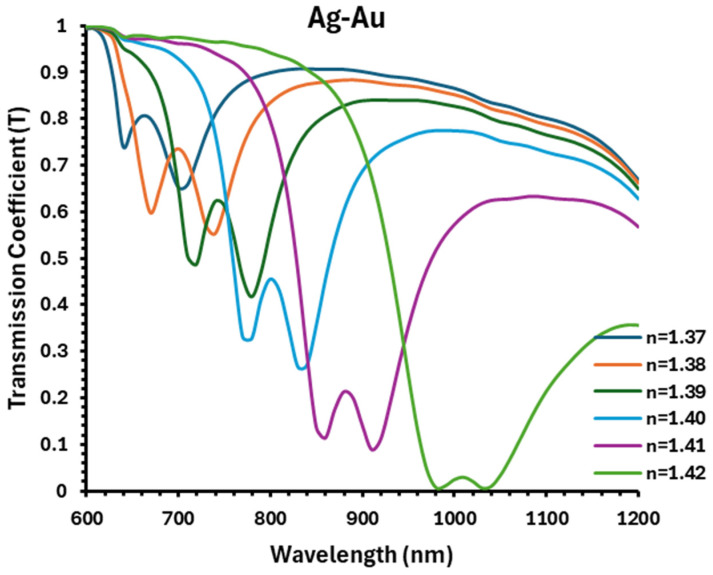
Transmission spectra for bimetallic asymmetric (Ag/Au) π-configuration sensor.

The transmission spectra of the Ag/Au π-sensor were simulated for analytes with refractive indices ranging from 1.37 to 1.42. Figure 3 shows two clearly resolvable attenuation dips across the range, each exhibiting a linear redshift as the analyte RI increases. The first dip, with narrow width, is obtained from the interaction of light with the Ag thin film, while the second peak, which is broader and shifts towards higher wavelengths, is obtained from the interaction of light with the Au thin film. The sensitivity of each sensing region can be calculated by linearly regressing the resonance wavelength shift ($∆\lambda_{res}$) against the refractive index change ($∆n_{a}$). The Ag-region exhibited a sensitivity of 12,700 nm/RIU, while the Au-region exhibited a lower sensitivity of 12,200 nm/RIU. This is due to Au’s broader resonance dip and more intrinsic loss. Crucially, the two sensitivities are orthogonal sensor outputs resulting from a single optical interrogation.

**Figure 4:**
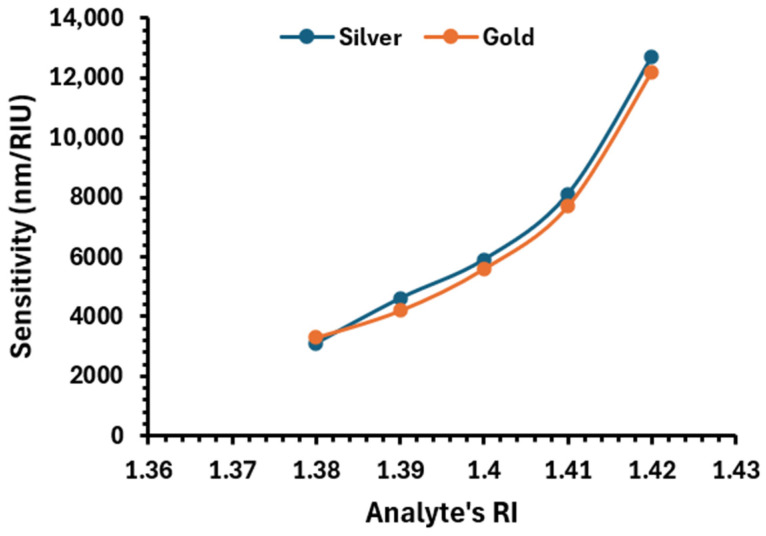
Plot of sensitivity against RI of bimetallic asymmetry π-configured sensor.

Figure 4 shows the plot of sensitivity against the analyte RI ranging from 1.37 to 1.42. The sensitivity obtained while varying the analyte RI is slightly greater when compared with the sensitivity of a single D-SPR gold or silver sensor for the same RI range (see Table 1).

This bimetallic asymmetry introduces material-dispersion encoded multiplexing, a new sensing paradigm. Unlike wavelength-division multiplexing, which uses separate light sources or complex grating structures [34], this method encodes multiple sensing regions into the device’s inherent material properties. The two resonance wavelengths are fixed by design through metal selection, and their independent shifts offer two simultaneous measurements of the surrounding dielectric environment. This investigation reveals that the π-configuration is not just a dual-channel sensor but also an integrated photonic sensor where strategic material asymmetry translates into spectrally resolved, multi-parameter output. It establishes the underlying proof that the design can accommodate independent sensing regions. The proposed sensor is designed for simultaneous dual-analyte detection from a sample, and each peak serves as an independent sensing channel.

### 3.3. Investigating π-Configuration Dual SPR Sensor Using Metal and Metal$-{TiO}_{2}$ Layers

**Figure 5:**
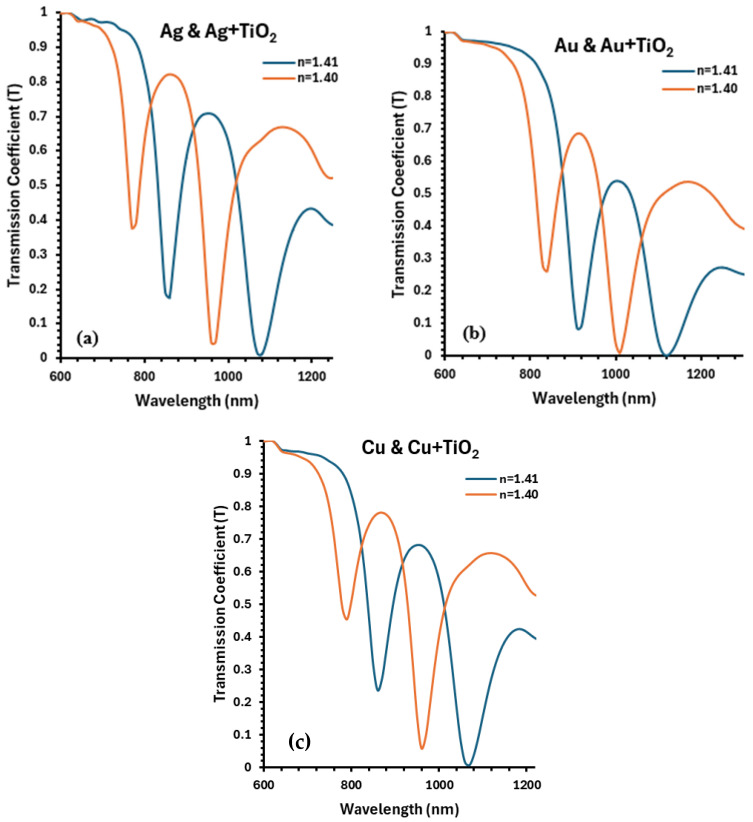
Transmission spectra for (**a**) Ag and Ag + ${TiO}_{2}$; (**b**) Au and Au + ${TiO}_{2}$; and (**c**) Cu and Cu + ${TiO}_{2}$ sensing regions.

The transmission spectrum of the π-configuration dual SPR sensor serves as the primary diagnostic tool for evaluating its performance, revealing critical insights into the plasmonic coupling efficiency at each sensing zone, and resonance peak separation (Δλ) for multi-analyte detection. This section combines theoretical modeling, finite-element simulations, and performance benchmarks to derive the spectral response of the dual-zone sensor using coupled-mode theory, quantify resonance wavelengths (λ_spr_) and sensitivities for Ag and Ag$-{TiO}_{2}$ films, and compare results with conventional single D-shaped SPR sensors. ${TiO}_{2}$ is a high-refractive-index dielectric with unique optoelectronic properties that enhance SPR performance by increasing evanescent field confinement at the metal–dielectric interface, boosting sensitivity. It is inert to oxidation and biofouling and improves the mechanical durability of metal thin films as an adhesive layer. The essence of using ${TiO}_{2}$ in π-configuration dual SPR sensing is to ensure spectral peak separation of the dips, enabling wavelength-division multiplexing (WDM). Figure 5 shows the transmission spectra for the metal and metal$-{TiO}_{2}$ sensing regions. The transmission spectrum for the Ag and Ag$-{TiO}_{2}$ sensing regions is shown in Figure 5a, while the Au and Au$-{TiO}_{2}$ and Cu and Cu$-{TiO}_{2}$ sensing regions are shown in Figure 5b and Figure 5c, respectively.

**Figure 6:**
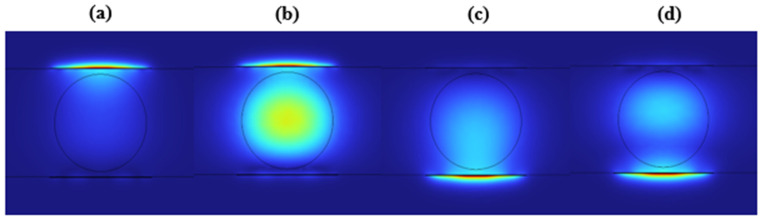
Electric field distribution for (**a**) Ag-region SPR mode; (**b**) Ag-region core mode; (**c**) Ag + ${TiO}_{2}$-region SPR mode; and (**d**) Ag + ${TiO}_{2}$-region core mode.

The metal/${TiO}_{2}$–analyte interface generates stronger spectral shift than the metal–analyte interface, increasing sensitivity. ${TiO}_{2}$’s lower loss coefficient minimizes plasmonic damping, reducing interference between zones. The metal thin film’s high plasmonic efficiency and ${TiO}_{2}$ RI engineering yields an optimized dual-region performance. Figure 6 illustrates the simulated electric field intensity distribution at the metal–dielectric and metal$-{TiO}_{2}$–dielectric interfaces of the π-configuration dual SPR optical fiber sensor. Figure 6a, b, c, and d show the Ag-region SPR mode, Ag-region core mode, Ag + ${TiO}_{2}$-region SPR mode, and Ag + ${TiO}_{2}$-region core mode, respectively. The figures confirm that two plasmonic modes are supported at the metal–analyte and metal$-{TiO}_{2}$–analyte interfaces, which contribute to two distinct dips in the transmission spectrum.

The metal $-{TiO}_{2}$ sensing region exhibits a longer red shift compared to the Ag-only zone due to the increased effective refractive index ($n_{eff}$). ${TiO}_{2}$ has a high RI (n ≈ 2.4–2.6) compared to the analyte (n = 1.37–1.42). When deposited on the metal thin film, ${TiO}_{2}$ displaces the lower-RI analyte near the metal surface, increasing the local $n_{eff}$ at the plasmonic interface. The SPR evanescent field penetrates further into the ${TiO}_{2}$ layer due to its higher n, causing increased field to overlap with the analyte and reduced plasmon momentum, requiring lower-energy (longer λ) photons for resonance.

**Figure 7:**
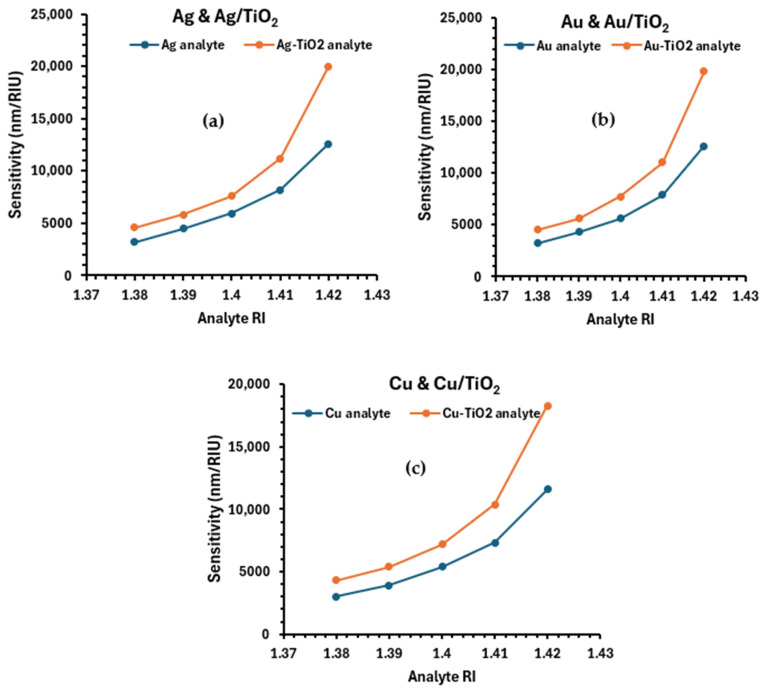
Wavelength sensitivity graph of (**a**) Ag and Ag$-{TiO}_{2}$; (**b**) Au and Au$-{TiO}_{2}$; and (**c**) Cu and Cu$-{TiO}_{2}$ sensing regions.

Figure 7 shows the wavelength sensitivity graph of the π-configuration dual SPR fiber optic sensor against the analyte RI. Figure 7a, Figure 7b, and Figure 7c show the sensitivity graph for Ag and Ag$-{TiO}_{2}$, Au and Au$-{TiO}_{2}$, and Cu and Cu$-{TiO}_{2}$ sensing regions, respectively.

From Figure 7a, silver and silver-${TiO}_{2}$ π-configuration dual sensors have the highest wavelength sensitivities of 12,600 nm/RIU and 20,000 nm/RIU, respectively, at an analyte RI of 1.42. Gold and gold-${TiO}_{2}$ sensors have wavelength sensitivities of 12,600 nm/RIU and 19,800 nm/RIU, respectively, while copper and copper-${TiO}_{2}$ sensors have wavelength sensitivities of 11,600 nm/RIU and 18,300 nm/RIU, respectively, for the same analyte RI. Silver had a slightly better sensitivity compared to gold due to the high plasma frequency, low optical losses, and better dielectric interactions. Silver has a high negative real permittivity (ε’), which enhances field confinement, and a low imaginary permittivity (ε’’), which reduces damping and minimizes losses, yielding deeper resonance dips and higher RI sensitivity. Ag$-{TiO}_{2}$ further enhances the field due to ${TiO}_{2}$’s high refractive index (*n* ≈ 2.6), which increases the effective RI contrast, thereby reducing quenching of plasmons. Gold exhibits broader resonance due to interband transitions, but at higher refractive indices and wavelengths (NIR), it has stronger negative real permittivity and a lower imaginary part, minimizing losses. Copper exhibits higher losses due to oxidation and defects but still finds applications where cost implications are a factor.

Our simulations assume ideal conditions: perfectly uniform thin films, atomically smooth metal–dielectric interfaces, perfect side-polished surfaces with exactly controlled residual cladding thickness (0.7 µm), without scattering losses from fabrication imperfections. This model also uses the Drude–Lorentz dispersion relations for metals without accounting for surface defects or grain boundaries. Furthermore, the FEM method in COMSOL solves Maxwell’s equations exactly for the idealized geometry, predicting the maximum theoretically achievable sensitivity, whereas experimental sensors inevitably suffer from coupling losses, modal noise, and imperfections introduced during polishing and thin-film deposition. Therefore, the reported sensitivity could be interpreted as the upper theoretical limit for this design under optimized conditions, which may be slightly larger compared to experimental values. The practical fabrication of the proposed π-configuration sensor will induce tolerances that may affect performance. Based on the experimental literature on side-polished and D-shaped fiber SPR sensors [17,20], typical variations include a residual cladding thickness of ± 0.1−0.2 μm, which can significantly shift resonance wavelengths; a metal film thickness non-uniformity of ± 5 nm, which may reduce sensitivity and broaden the FWHM; and surface roughness of the polished fiber causing an increase in scattering losses and dip depth reduction.

**Figure 8:**
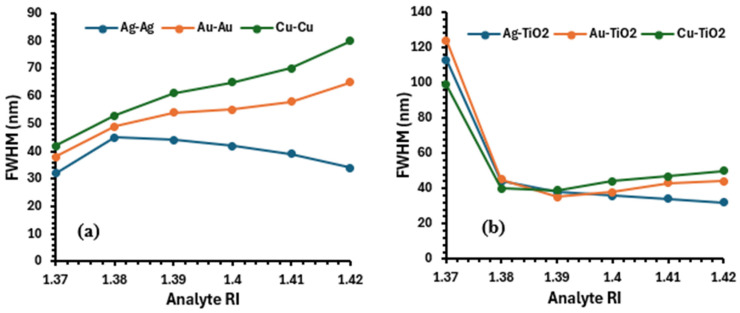
FWHM against RI graph of (**a**) metal–metal regions and (**b**) metal–metal/${TiO}_{2}$ regions.

Figure 8a and Figure 8b are the graphs of FWHM against the analyte RI for metal–metal and metal–metal/${TiO}_{2}$ sensors, respectively. FWHM is the spectral width of an SPR peak at half its maximum intensity. A narrower FWHM indicates a sharper resonance peak, leading to a higher resolution in detecting small RI changes. A broader FWHM reduces resolution because small spectral shifts become harder to distinguish from noise [35]. Ag–Ag clearly showed a smaller FWHM compared to Au–Au and Cu–Cu for the metal–metal sensing regions, while Ag–Ag/${TiO}_{2}$ showed a smaller FWHM at a higher RI compared to the Au–Au/${TiO}_{2}$ and Cu–Cu/${TiO}_{2}$ regions, indicating a sharper resonance dip. For the π-configuration sensor with distinct peaks, a narrow FWHM helps to clearly resolve them. An overlap in broad peaks due to large FWHM can occur, reducing the ability to distinguish between two analytes or sensing regions.

Table 2 shows the exact resonant peak positions for all three material cases (Ag, Au, Cu) and their corresponding metal–TiO_2_ configurations across the entire refractive index range of 1.37–1.42, while Table 3 shows a comparison of the difference between the metal–analyte peak wavelength $\left(∆\lambda_{SPR1}\right)$ and the metal$-{TiO}_{2}$–analyte peak wavelength $\left(∆\lambda_{SPR2}\right)$.

In the π-configuration dual SPR sensor, the spectral separation $\left(∆\lambda_{SPR}\right)$ between the resonance dips of the two sensing zones is critical for enabling multi-analyte detection, minimizing potential crosstalk, and optimizing sensitivity. The ability to resolve two distinct peaks depends on material-induced shifts and coupling conditions. A sufficiently large $∆\lambda_{SPR}$ ensures that each peak can be independently tracked for RI changes in two different analytes.

The Rayleigh Criterion states that two peaks are resolvable if ${∆\lambda }_{SPR}$ ≥ FWHM (full width at half maximum) of the narrower peak [36]. If $∆\lambda_{SPR}$ is too small (<50 nm), the evanescent field overlaps between zones cause peak merging (loss of dual-analyte capability) and inaccurate sensitivity calculations (apparent shift ≠ true RI change). For this sensor design, $∆\lambda_{SPR}$ > 125 nm was achieved for all refractive index ranges (n = 1.37–1.42), thereby minimizing potential crosstalk and ensuring each peak in the spectrum is easily identified. Table 4 shows a comparison of the novel π-configuration SPR-based optical fiber sensor utilizing metal + metal/${TiO}_{2}$ thin films with existing designs reported in the literature.

## 4. Conclusions

This study successfully designed, modeled, and characterized a novel π-configuration dual SPR sensor based on a single D-shaped optical fiber with opposing side-polished regions functionalized with Ag/Au/Cu and Ag/Au/Cu$-{TiO}_{2}$ thin films. Through comprehensive finite-element simulations using COMSOL Multiphysics and theoretical analysis, we demonstrated that this architecture enables high-sensitivity, multi-analyte detection while minimizing crosstalk between sensing zones, a critical limitation in conventional SPR designs. Geometric isolation and spectral resolvability are the two factors that constitute justification for crosstalk minimization in this design.

The investigation of the π-configuration sensor featuring a bimetallic asymmetry (Ag/Au) design produced two resolvable resonance dips, at 983 nm (Ag) and 1034 nm (Au), with sensitivities of 12,700 nm/RIU and 12,200 nm/RIU, respectively, for an analyte RI of 1.42. The integration of metal–dielectric (Ag$-{TiO}_{2}$) in one sensing region induced a significant red shift of 294 nm (982 nm vs. 1276 nm), enabling wavelength-division multiplexing (WDM) for simultaneous dual-analyte detection. Although silver exhibits strong absorption around 400 nm due to interband transitions, the SPR wavelength is not fixed but depends critically on the dielectric environment and the waveguide geometry. At near-infrared wavelengths, silver’s permittivity is dominated by free-electron behavior with low imaginary losses, allowing phase-matching at wavelengths far from the 400 nm interband region. ${TiO}_{2}$’s high refractive index (n ≈ 2.6) further increased the local effective RI at the metal–dielectric interface, requiring a longer wavelength to satisfy the phase-matching condition, hence the shift from 982 nm (Ag–analyte) to 1276 nm (Ag–TiO_2_–analyte). TiO_2′_s high RI also enhanced the local evanescent field, boosting sensitivity from 12,600 nm/RIU in the Ag–analyte region to 20,000 nm/RIU in Ag–TiO_2_–analyte region. ${TiO}_{2}$ coating further improved stability by mitigating Ag oxidation, a key advantage over Cu-based sensors. The >125 nm peak separation resolved two distinct analytes, eliminating the need for complex multi-core fibers or sensor arrays, reducing system cost and complexity.

This work establishes the π-configuration dual SPR sensor as a compact, high-performance platform for multiplexed sensing, addressing key limitations of traditional SPR systems. By combining geometric innovation, hybrid material engineering, and crosstalk-optimized design, this research opens new avenues for next-generation optical biosensors in healthcare, environmental monitoring, and industrial applications. This research design has potential for real-time, label-free monitoring of biomarkers, and multiplexed detection of heavy metals or organic pollutants in water. While we have not yet experimentally realized this specific sensor, every individual fabrication step, including dual-side polishing and metal and dielectric deposition, has been validated in previous work on D-shaped and side-polished fiber SPR sensors. Future work could include the experimental validation of the sensor in real biofluid samples, integration with microfluidics for lab-on-a-chip applications, and exploration of 2D materials such as graphene or MoS_2_ for further sensitivity enhancement.

## Figures and Tables

**Table 1:** Comparison of sensitivities for π-configuration and D-shaped SPR sensors.

RI	Sensitivity (nm/RIU)					
	π-Configuration Dual SPR Sensor			D-Shaped SPR Sensor		
	Ag–Ag	Au–Au	Cu–Cu	Ag	Au	Cu
1.37	-	-	-	-	-	-
1.38	3300	3300	3000	1500	3200	2200
1.39	4400	4300	3900	4200	4200	3700
1.40	5900	5600	5400	5800	5500	5300
1.41	8100	7900	7300	8000	7800	7200
1.42	12,500	12,400	11,400	12,300	12,200	11,300

**Table 2:** SPR resonant wavelengths for the three material cases (Ag, Au, and Cu).

Analyte RI	Peak Wavelength for Metal and Metal–TiO_2_ Sensor (nm)					
	Ag	Ag–TiO_2_	Au	Au–TiO_2_	Cu	Cu–TiO_2_
1.42	982	1276	1040	1317	977	1249
1.41	856	1076	914	1119	861	1066
1.40	774	964	835	1009	788	962
1.39	715	888	779	932	734	890
1.38	670	830	736	876	695	836
1.37	638	784	704	831	665	793

**Table 3:** Spectral separation for π-configuration metal$-{TiO}_{2}$ sensors.

Analyte RI	Peak Wavelength Separation (${\Delta \lambda }_{SPR}$) for Metal–TiO_2_ Sensor (nm)		
	Ag–TiO_2_	Au–TiO_2_	Cu–TiO_2_
1.42	294	277	272
1.41	220	205	205
1.40	190	173	174
1.39	173	153	156
1.38	160	140	141
1.37	146	127	128

**Table 4:** Comparison of the π-configuration SPR-based optical fiber sensor with existing sensors reported in the literature.

Ref	Sensor Configuration	Sensing Material	RI Range	Wavelength Sensitivity (nm/RIU)	FOM	Resolution (RIU^−1^)
[37]	Dual-core D-shaped PCF	Silver	1.35–1.5	3400	-	2.94 × 10^−5^
[38]	Dual-symmetrical D-shaped	Gold	1.21–1.22	5000	-	-
[39]	Dual-channel PCF	Gold	1.33–1.40	11,600	-	8.62 × 10^−6^
[40]	Dual-core D-shaped	Gold	1.33–1.44	17,000	320	5.88 × 10^−6^
	π-config SPR	Silver	1.37–1.42	12,600	394	7.94 × 10^−6^
This work	π-config SPR	Ag/TiO_2_	1.37–1.42	20,000	625	5.0 × 10^−6^

## Data Availability

The data supporting the findings of this study are available from the corresponding author upon reasonable request.
